# Geothermometry of calcite spar at 10–50 °C

**DOI:** 10.1038/s41598-024-51937-4

**Published:** 2024-01-18

**Authors:** Gabriella Koltai, Tobias Kluge, Yves Krüger, Christoph Spötl, László Rinyu, Philippe Audra, Charlotte Honiat, Szabolcs Leél-Őssy, Yuri Dublyansky

**Affiliations:** 1 Institute of Geology, University of Innsbruck, Innrain 52, 6020 Innsbruck, Austria; 2 Institute of Environmental Physics, Heidelberg University, Im Neuenheimer Feld 229, 69120 Heidelberg, Germany; 3 Chair of Geochemistry and Economic Geology, Karlsruhe Institute of Technology, Adenauerring 20B, 76131 Karlsruhe, Germany; 4 Department of Earth Science, University of Bergen, Allégaten 41, 5007 Bergen, Norway; 5 Isotope Climatology and Environmental Research Centre (ICER), HUN_REN Institute for Nuclear Research (Atomki), Bem tér 18/C, 4026 Debrecen, Hungary; 6 Polytech’Lab-UPR 7498, University of Côte d’Azur, 930 Route des Colles, Sophia-Antipolis, 06903 Nice, France; 7 Department of Physical and Applied Geology, Eötvös Loránd University, Egyetem tér 1-3, 1053 Budapest, Hungary

**Keywords:** Environmental sciences, Hydrology, Geochemistry

## Abstract

Carbonate geothermometry is a fundamental tool for quantitative assessment of the geothermal and geochemical evolution of diagenetic and hydrothermal systems, but it remains difficult to obtain accurate and precise formation temperatures of low-temperature calcite samples (below ~ 40 to 60 °C). Here, we apply three geothermometry methods (∆_47_-thermometry, nucleation-assisted fluid inclusion microthermometry—hereafter NA-FIM—and oxygen isotope thermometry) to slow-growing subaqueous calcite spar samples to cross-validate these methods down to 10 °C. Temperatures derived by NA-FIM and Δ_47_-thermometry agree within the 95% confidence interval, except for one sample. Regression analyses suggest that the real uncertainty of ∆_47_-thermometry exceeds the 1 SE analytical uncertainty and is around ± 6.6 °C for calcite spar that formed at 10–50 °C. The application of δ^18^O thermometry was limited to a few samples that contained sufficient primary fluid inclusions. It yielded broadly consistent results for two samples with two other geothermometers, and showed higher temperature for the third spar. We also found that calcite with steep rhombohedral morphologies is characteristic of low temperatures (11–13 °C), whereas blunt rhombohedra prevail in the 10–29 °C domain, and the scalenohedral habit dominates > 30 °C. This suggests that the calcite crystal morphology can be used to qualitatively distinguish between low- and higher-temperature calcite.

## Introduction

### Low-temperature geothermometry of calcite

Many processes in the shallow Earth’s crust involve the movement of water and water–rock interactions at low temperatures (10–50 °C), including the formation of new minerals. Examples include diagenetic reactions (e.g., precipitation of cements in sediments, dolomitization, dedolomitization), formation of low-temperature hydrothermal minerals, and the deposition of speleothems in caves. Because of its relatively high solubility in the near-surface environment, the dual control on its solubility (temperature, hereafter T, and *p*CO_2_), and the ubiquity of carbonate-bearing rocks, calcite is one of the most common minerals involved in these processes^1,2^. Its occurrence in various types of rocks therefore provides important insights into water–rock interactions in the geological past and the composition of the paleowaters involved in these reactions (e.g.^3–5^). Most importantly, however, calcite is an excellent candidate for quantitively determining the ambient thermal conditions at the time of mineral formation.

Fluid inclusion microthermometry (hereafter FIM) has long been the geothermometrical method of choice for T > ca. 50 °C^6,7^. This method exploits the fact that during crystal growth, crystals commonly trap small amounts of mineral-forming fluid in tiny vacuoles, forming fluid inclusions (FIs). Fluid inclusions are nearly isochoric systems, implicating that the fluid density remains constant with changing temperature while pressure changes along density-specific isochores. An initially single-phase liquid inclusion that formed at elevated temperature and subsequently cooled to room temperature typically features a vapour bubble that forms by spontaneous nucleation at negative fluid pressure (tensile stress) in the metastable liquid state of the inclusion^8^. Upon subsequent heating, the liquid phase expands at the expense of the vapor bubble and finally the inclusion homogenizes again to the liquid phase. The temperature at which the vapour bubble vanishes is called liquid–vapor homogenization temperature (*T*_*h*_) and provides an estimate of the *minimum* formation temperature of the inclusion, and in case of primary FIs, of the confining host mineral^6,7^. Low-T FIs, in contrast, are typically single-phase liquid at room temperature and spontaneous nucleation of the vapor bubble fails upon cooling due to long-lived metastability of the liquid state of water, which previously made measurements of liquid–vapor *T*_*h*_ in these inclusions impossible. Today, these limitations can be overcome by means of ultra-short laser pulses that stimulate vapor-bubble nucleation in the metastable liquid and thus convert the inclusions into a stable liquid–vapor equilibrium state,—a precondition for subsequent measurements of *T*_*h*_^9^. Nucleation-assisted fluid inclusion microthermometry (hereafter NA-FIM) enables microthermometric analyses of FIs that formed at temperatures as low as 9 °C^10^ (depending on the size of the inclusions).

In addition to FIM, several other methods have been used to determine the formation temperature of calcite, notably oxygen isotope thermometry^11,12^ (hereafter OIT) and clumped isotope thermometry^13–15^ (hereafter ∆_47_-thermometry). OIT exploits the temperature-dependent fractionation of oxygen isotopes between water and calcite. This method requires the independent knowledge of the isotopic composition of both calcite and the mineral-forming water (δ^18^O_c_ and δ^18^O_w_). In many cases δ^18^O_w_ is unknown and has to be assumed using other constraints. Technical improvements^16,17^ allow in some cases to directly measure the stable isotopic composition of FI water.

Clumped isotope thermometry measures the “clumping” of the heavy isotopes ^13^C and ^18^O into bonds in calcite, expressed as Δ_47_ value, which depends on temperature. This method does not require a priori knowledge of the δ^18^O composition of the mineral-forming water; it does require, however, that carbonate precipitation occurred under isotopic equilibrium^13–15^.

In this study we performed a cross-comparison of NA-FIM, ∆_47_-thermometry and OIT, using natural samples that formed in the range of 10–50 °C. Each of the three methods has inherent limitations and caveats, which affect precision and accuracy of the obtained temperatures and may affect the applicability of these methods in some situations.

### Study design and sample selection

We used NA-FIM as the primary method for comparison with ∆_47_-thermometry and OIT-derived temperatures obtained on the same samples. This decision was based on the fact that FIM (including NA-FIM) has an internal ‘data quality check’ that allows the reliability of the results to be evaluated based on the temperature variation obtained on individual FI assemblages (FIAs; groups of petrographically associated FIs that were likely entrapped at the same time). The currently accepted criterion is that 90% of the *T*_*h*_ obtained from a FIA should fall within a 10 °C interval^7^.

To minimize potential biases due to isotopic disequilibrium that may affects OIT and ∆_47_-thermometry, all measurements were made on coarsely-crystalline euhedral calcite crystals (spar) that formed in subaqueous environments and at very likely very slow growth rates (e.g.^18^ and Sample selection in the Supplementary Information). Samples of this study were collected from caves, vugs and veins in carbonate rocks in Hungary, Austria, France, and Kyrgyzstan (Table S1). Only occurrences lacking evidence of weathering and direct exposure to elevated temperatures at the surface were sampled.

FI petrography was performed on all samples (Supplementary Information, Fig S1). Well-defined primary FIAs were identified in 11 calcite spars, which were retained for further analyses (Table S2). On all these samples, formation temperatures were determined by NA-FIM and ∆_47_-thermometry. OIT could be applied only on three samples that contained sufficient primary and only few secondary FIs.

Stable isotope analyses of calcite (δ^18^O_c_ and δ^13^C_c_) along the crystal growth axis were performed to provide a semi-quantitative assessment of possible temperature variations and/or changes in the isotopic composition of the paleowater during crystal growth.

## Results

Temperatures obtained by the different methods on the same samples are summarized in Table 1.

**Table 1:** Comparison of temperatures obtained by NA-FIM, Δ_47_ and OIT methods.

Sample ID	NA-FIM			Δ_47_				OIT		
	Mean T_*h∞*_ (°C)	SE (°C)	Number of FIs	Δ_47_ ± 1 SE	T (°C)	SE (°C)	Number of replicates	T (°C)	SD (°C)	Number of replicates
ESZ-2-1 core	13.7	0.5	18	0.636 ± 0.009	11.7	2.6	15			
ESZ-2-2 core2	12.9	0.5	16	0.639 ± 0.006	10.9	2.7	17			
ESZ-3	11.0	0.5	10	0.637 ± 0.006	11.4	2.6	15	10.5	3.8	9
RKC-2	12.4	0.5	15	0.625 ± 0.009	14.9	3.2	15			
BEQ-9	13.3	1.6	10	0.639 ± 0.009	10.8	3.2	15			
FEC-1	28.5	0.9	16	0.596 ± 0.011	23.5	4	3			
NKQ4-3-1 core	32.7	0.6	10	0.546 ± 0.009	42.5	4.3	14			
NKQ4-3-2 rim	20.4	0.5	14	0.580 ± 0.008	29.7	3.0	16			
NKQ4-4	25.3	0.5	10	0.583 ± 0.009	28.6	3.2	17			
SB-10	16.5	0.7	12	0.605 ± 0.009	21.3	3.5	14	23.1	1.7	5
SUR-13	41.0	0.7	15	0.559 ± 0.007	37.7	3.6	14			
SPA-147	31.4	0.6	13	0.569 ± 0.007	33.9	3.4	14	44.8	4.6	4
PIG-1	19.6	0.5	19	0.599 ± 0.009	23.1	3.5	14			

NA-FIM temperatures are reported as standard error of the mean (1 SE) and also account for the overall analytical precision. Δ_47_ values are reported relative to (I)-CDES-90. Clumped isotope temperatures were calculated after Anderson et al.^19^and 1 SE errors include the uncertainty of the calibration. δ^18^O_w_–δ^18^O_c_ temperatures were calculated using the equation of Daëron et al.^22^.

### NA-FIM

**Figure 1:**
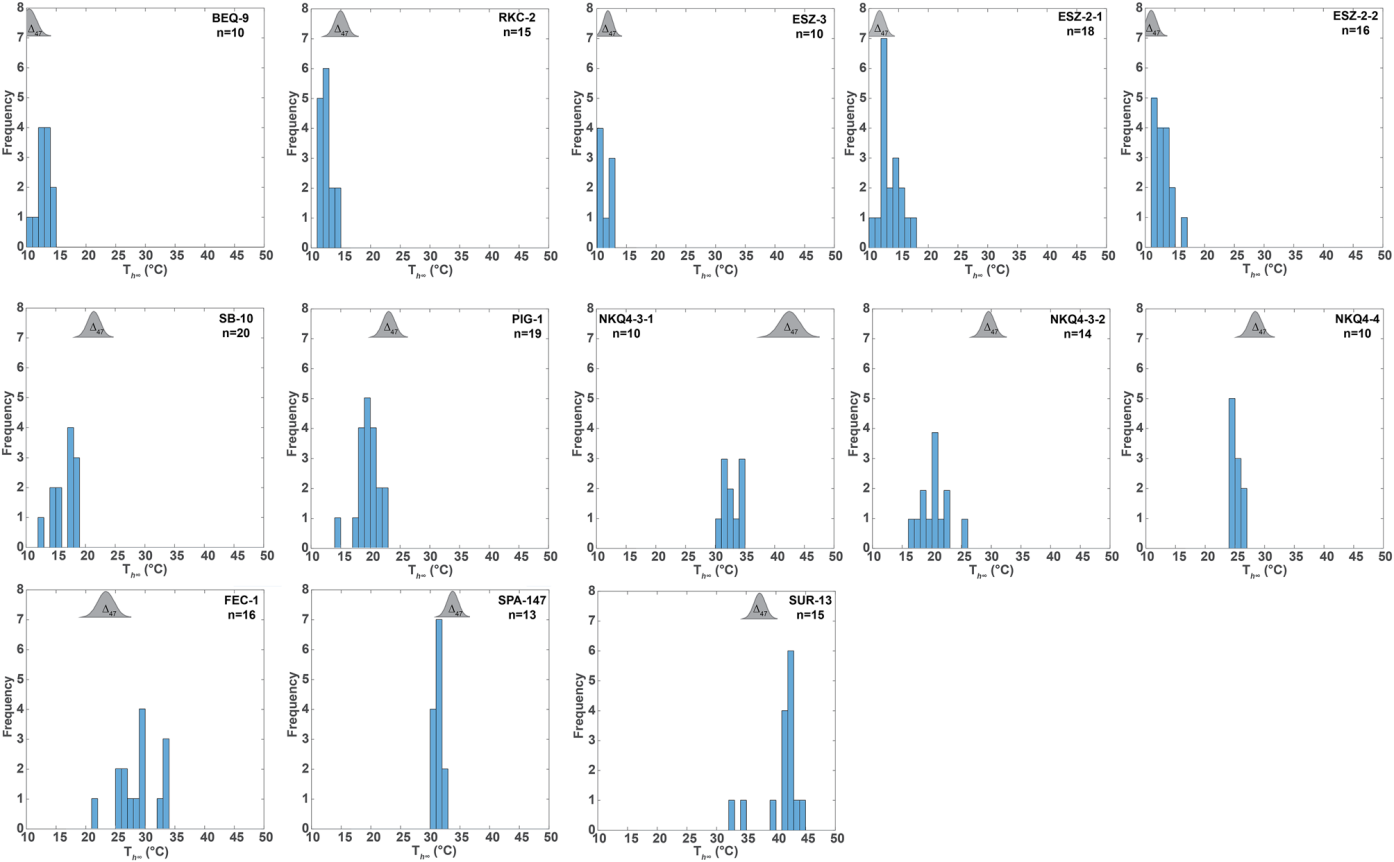
Bubble-size corrected homogenization temperatures (T_*h∞*_) of initially single-phase fluid inclusions in calcite spar samples (bin size 1 °C). Grey shaded areas show the probability density distribution of the Δ_47_ data (± 1 SE).

Mean values of surface tension-corrected homogenization temperatures (T_*h∞*_)^10^ obtained from primary, initially single-phase liquid inclusions range from 11 to 41 °C (Table 1). Between 10 and 19 inclusions per sample were analyzed. Variations of T_*h∞*_ values within individual FIAs and across different FIAs in the same samples were mostly well below 10 °C (between 1.4 and 8.8 °C, Fig. 1), with standard errors (1 SE) ranging between 0.5 and 1.6 °C. The results demonstrate the high precision that can be achieved with NA-FIM. Ice melt temperatures indicate that the salinity of the FIs are between 0.5 to 0.9 wt.% NaCl equivalent (Table S3).

### Carbonate isotope analyses (∆_47_, δ^18^O_c_, δ^13^C_c_)

The Δ_47_ measurements were performed on calcite powders drilled at the same locations on the crystals where the NA-FIM data were obtained. Δ_47_ values range from 0.639 to 0.546‰ (relative to the (I)-CDES-90 reference frame) with standard errors of the mean (1 SE) of 0.006 to 0.011 ‰ (Tables 1 and 2). This translates into analytical temperature uncertainties between 1.5 and 3.8 °C (1 SE). The Δ_47_ values were converted into temperatures using the calibration equation of Anderson et al. ^19^ (Tables 1 and 2), resulting in formation temperatures between 10.8 and 42.5 °C and uncertainties of 2.6 to 4.3 °C which include the uncertainty of the calibration equation of 1.9–2.4 °C (95% CL) (Table 1).

**Table 2:** Comparison of Δ_47_ temperatures obtained in the laboratories of Heidelberg and Debrecen.

Sample ID	Heidelberg				Debrecen					Combined data			
	Δ_47_ ± 1 SE	T (°C)	Analytical SE (°C)	Number of replicates	Δ_47_ ± 1 SE	T (°C)	Analytical SE (°C)	95% CL (°C)	Number of replicates	Δ_47_ ± 1 SE	T (°C)	Analytical SE (°C)	SE (°C)
ESZ-2-1 core	0.641 ± 0.013	10.2	4.3	3	0.632 ± 0.009	12.7	2.6	5.8	12	0.636 ± 0.009	11.7	2.6	3.2
ESZ-2-2 core2	0.653 ± 0.015	6.8	4.8	4	0.632 ± 0.007	12.7	2.7	4.3	12	0.639 ± 0.006	10.9	1.9	2.7
ESZ-3	0.660 ± 0.012	4.8	4.0	4	0.626 ± 0.006	14.7	1.8	3.9	11	0.637 ± 0.006	11.4	1.7	2.6
RKC-2	0.632 ± 0.005	12.9	2.4	3	0.613 ± 0.009	18.7	2.8	6.3	12	0.625 ± 0.009	14.9	2.6	3.2
BEQ-9	0.653 ± 0.027	6.8	8.2	3	0.634 ± 0.009	12.2	3.3	6.0	12	0.639 ± 0.009	10.8	2.6	3.2
FEC-1	0.596 ± 0.011	24.3	3.8	3									
NKQ4-3-1 core	0.573 ± 0.021	32.3	6.5	2	0.529 ± 0.010	49.9	4.5	9.0	12	0.546 ± 0.009	42.5	3.6	4.3
NKQ4-3-2 rim	0.587 ± 0.006	27.3	2.6	4	0.571 ± 0.008	33.0	3.0	6.5	12	0.580 ± 0.008	29.7	2.4	3.0
NKQ4-4	0.588 ± 0.006	27.0	2.6	5	0.577 ± 0.009	30.9	3.1	6.9	12	0.583 ± 0.009	28.6	2.6	3.2
SB-10	0.643 ± 0.002	9.6	2.0	2	0.584 ± 0.007	28.4	3.3	6.1	12	0.605 ± 0.009	21.3	2.9	3.5
SUR-13	0.563 ± 0.008	36.0	3.0	2	0.556 ± 0.009	38.7	3.8	7.3	12	0.559 ± 0.007	37.7	2.8	3.6
SPA-147	0.559 ± 0.006	37.6	3.0	2	0.574 ± 0.008	31.9	3.4	6.3	12	0.569 ± 0.007	33.9	2.5	3.4
PIG-1	0.581 ± 0.011	29.5	3.8	2	0.612 ± 0.006	19.2	3.5	6.5	12	0.599 ± 0.009	23.1	2.8	3.5

Clumped isotope temperatures were calculated after Anderson et al.^19^.

Δ_47_ values measured in Heidelberg are reported relative to CDES-90 (carbon dioxide equilibrium scale at 90°C), while data measured in Debrecen are reported relative to I-CDES. The ∆_47_ values of the two laboratories were combined by calculating an error-weighted mean of the (I)-CDES-90 values for each sample. The ∆_47_ uncertainty of the combined data is given as standard error (1 SE), which includes the temperature uncertainty of the calibration line of Anderson et al.^19^.

The results of δ^18^O_c_ and δ^13^C_c_ transects across individual calcite crystals are shown in Fig. S2. In three of the samples, δ^18^O_c_ variations exceed 2‰ (SUR-13, SPA-147, and NKQ4-3), while four samples exhibit changes in δ^13^C_c_ of more than 3‰ (RKC-2, ESZ-3, SUR-13, and NKQ4-3). All other calcite spars indicate only minor variations in stable isotopic composition, indicating relatively stable growth conditions.

### Fluid inclusion water isotopes (δ^2^H_w,_ δ^18^O_w_) and isotope geothermometry

Of the six samples that were initially selected for water isotope analysis based on FI petrography (containing predominantly primary FIs), only three (ESZ-3, SB-10, and SPA-147) contained sufficient water for stable isotope analysis (0.1 to 0.9 µL/g). Water was released from the FIs by crushing 0.9 to 1.4 g of calcite. In total 18 measurements were performed (4 to 9 replicates per sample; Table S4). Measured δ^18^O_w_ yielded 1 SD between 1.3 and 2.4 ‰, leading to temperature uncertainties of ~ 6 to 14 °C. We therefore calculated δ^18^O_w_ from the measured δ^2^H_w_, using the modern Local Meteoric Water Lines^20,21^. Measured and calculated FI oxygen isotope values agree within the 1 SD (Table S4). Given the lower uncertainty of δ^2^H_w_, we used the calculated δ^18^O_w_ values along with the δ^18^O_c_ values measured on the crushed samples to calculate calcite formation temperatures applying the equation of Daëron et al.^22^. We chose this calibration because it is based on very slow-growing calcite from two subaqueous environments (Devils Hole, USA, and Corchia Cave, Italy), similar to the studied calcite spars. Mean temperatures derived from the replicate measurements are given in Table 1. Uncertainties include the standard deviation of δ^2^H_w_ obtained from replicate measurements (Table S4) as well as the analytical uncertainty of δ^18^O_c_ measurements. For formation temperatures calculated by other equations (e.g., Kim and O’Neil^23^; Tremaine et al.^24^) we refer the reader to the Supplementary Information (Table S6).

## Discussion

### Stalagmites, calcite spar and low-temperature geothermometry

An earlier attempt to systematically compare methods of low-T calcite geothermometry was reported by Meckler et al.^25^. This study was performed on two stalagmites formed in a limited temperature range of ca. 18–24 °C. Deposition of calcite in stalagmites occurs under subaerial conditions and at atmospheric pressure, therefore calcite precipitation is controlled by degassing of CO_2_ from a thin water film. The isotopic composition of calcite in such vadose settings may reflect both primary depositional parameters (i.e., δ^18^O of meteoric water, cave air temperature and δ^13^C of the carbonate bedrock and the soil-derived CO_2_ dissolved in water), and possible kinetic processes during precipitation (e.g., Rayleigh distillation of the HCO_3_^−^ reservoir during degassing of CO_2_ or evaporation^26^).

Given its depositional setting, stalagmite calcite appears to be an ideal candidate to apply the NA-FIM method (provided that T is higher than 9 °C). Calcite deposition at atmospheric pressure eliminates the need for pressure correction and the measured T_*h∞*_ values faithfully reflect the formation T. Dripstone caves, however, are less suitable for the application of isotope-based thermometry (Δ_47_ and OIT), as isotopic equilibrium during calcite precipitation cannot be assumed, and must always be ascertained by replication^27^. Meckler et al.^25^. demonstrated that the empirical speleothem-based fractionation equation of Tremaine et al.^24^ compensated for an average disequilibrium and thus provided accurate temperature estimates for the two stalagmites, whereas other studies used a cave-specific Δ_47_-thermometer calibration based on modern-day speleothems to correct for disequilibrium isotope fractionation^28,29^.

In contrast to stalagmites, calcite spar forms in phreatic settings, typically at elevated temperatures. The main mechanism controlling the deposition of calcite in such environments is the very slow degassing of CO_2_, caused by slow upwelling (decreasing pressure) and slow cooling of the groundwater^18,30, 31^. Slow CO_2_ degassing in deep-seated conditions within large groundwater bodies greatly reduces the possibility of kinetic processes and renders their distorting effects on isotopic systematics of the depositing spar negligible.

In most cases, the depth (and, respectively, P) of calcite spar precipitation is not known, and can only be assessed indirectly. From geochemical considerations, precipitation of low-T hydrothermal calcite is unlikely to occur at depths exceeding 300 m (at hydrostatic pressure conditions^30^). T_*h*_ values of FIs provide a *minimum* estimate of the temperature of formation, and a pressure correction is required to compensate for the elevated P at the site of calcite precipitation. All our samples are from competent carbonate rocks and therefore we assume a hydrostatic pressure gradient (~ 10 MPa/km). As the pressure correction is ca. 1 °C/MPa, the T_*h*_ of calcite formed in deep phreatic conditions may underestimate the formation T by up to ca. 3 °C.

In summary, calcite spar is an excellent candidate for comparing isotope-based thermometry (Δ_47_ and OIT) and NA-FIM, with a small bias towards lower temperatures in the latter method.

### Temperature of formation and calcite crystal morphology

The calcite crystal morphology reflects the environmental conditions and the composition of the water. Although the controlling parameters are numerous (T, *p*CO_2_, trace elements, etc.), empirical studies of natural systems revealed a more or less systematic evolution of crystal morphology^32–34^. Calcite crystals forming in ambient-temperature caves have a characteristic rhombohedral (with dominant steep rhombohedra) and, less commonly, scalenohedral morphologies^35^. Among our samples, we observed steep rhombohedral, blunt rhombohedral and scalenohedral crystal morphologies (Table S1). The steep rhombohedral calcites yielded the lowermost temperatures of formation (NA-FIM; 11–13 °C); crystals with blunt rhombohedral morphologies formed in a relatively wide temperature interval of 12–29 °C; and scalenohedral calcites formed between > 30 °C (Fig. S3). Thus, we propose that the crystal morphology (rhombohedral vs. scalenohedral) may be used to discriminate between lower- and higher-T varieties of calcite spar. This is consistent with data from deep carbonate aquifers^36^.

### Comparison of NA-FIM and Δ_47_ temperatures

**Figure 2:**
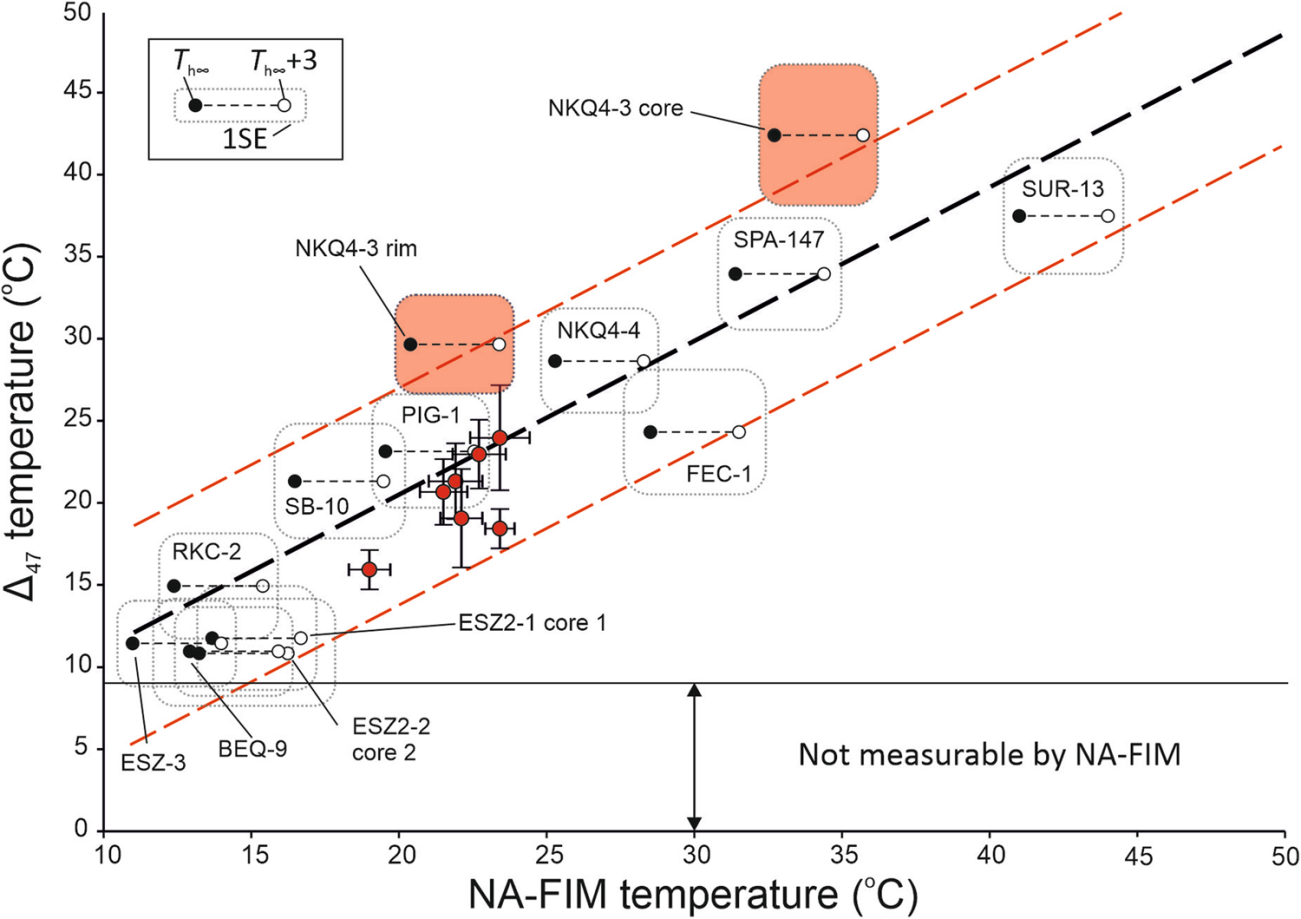
Comparison of NA-FIM and Δ_47_ temperatures (using the calibration by Anderson et al.^19^) obtained on the same samples. Black circles show measured T_*h∞*_; open circles show T_*h∞*_ + 3°C (corrected for 300 m of hydrostatic pressure). Red circles mark stalagmite data from Meckler et al.^25^ Δ_47_ temperatures were re-calculated using the calibration of Anderson et al.^19^. Dashed black line shows regression line; dashed red lines correspond to the 95% probability interval. Errors are shown by transparent boxes (1 SE, Table 1). Samples NKQ4-3-1 and NKQ4-3-2 (in orange) were excluded from the regression.

The T_*h∞*_ data indicate that the studied calcite samples precipitated between ca. 11 and 44 °C. To calculate Δ_47_ temperatures we applied the unified regression equation of Anderson et al.^19^. A comparison of the NA-FIM and Δ_47_ temperatures is shown in Fig. 2. The regression line has a slope close to unity (0.90), an intercept of 1.04 °C and a SE of 4.7 °C, yielding a 95% confidence interval of ± 9.4 °C.

As discussed above, data obtained using NA-FIM may slightly underestimate temperatures (by < 3 °C) due to (possibly) elevated hydrostatic pressure during spar formation. Δ_47_ temperatures, in contrast, are considered as true calcite formation temperatures. Assuming that the Δ_47_ temperatures are accurate, they are expected to be equal to or higher than T_*h∞*_.

One sample (NKQ4-3) yielded Δ_47_ temperatures significantly higher than T_*h∞*_. Two growth phases were identified in this sample: an early (NKQ4-3-1, core) and a later stage (NKQ4-3-2, rim), with temperature differences between the two methods of 9.8 ± 4.9 °C and 9.3 ± 3.5 °C, respectively (Table 1, Fig. 2). If both temperature estimates are accurate, this difference may be used to estimate the fluid trapping pressure. The 9.8 ± 4.9 °C and 9.3 ± 3.5 °C difference therefore suggests calcite precipitation at a minimum depth of ca. 490 m. The scenario of calcite forming at such great depth, however, is unlikely, because calcite deposition is not expected at depths greater than about 300 m^30^. However, calcite spar formation at a depth of ~300 m cannot be ruled out and would be consistent with regional groundwater temperatures (~ 40 °C at 500 m depth^37^). The similar temperature offset between NA-FIM and Δ_47_-temperatures in the two growth phases of NKQ4-3 may indicate non-equilibrium isotope fractionation or its combination with calcite deposition at a maximum depth of about 300 m. NKQ4-4 represents the next calcite generation in this mineralization sequence, for which the results of the two geothermometry methods agree (Table 1).

### Comparison of NA-FIM and OIT temperatures

We applied δ^18^O thermometry to three samples (ESZ-3, SB-10 and SPA-147). The small number of samples is related to two factors: (1) five spars contained secondary fluid inclusions and thus the isotope values measured on FI waters are not representative of the paleowater, and (2) three of the samples did not contain enough water for reliable analyses. Although our assessment of OIT vs. NA-FIM thermometry is based on only three samples, these observations may have important implications for future studies.

**Figure 3:**
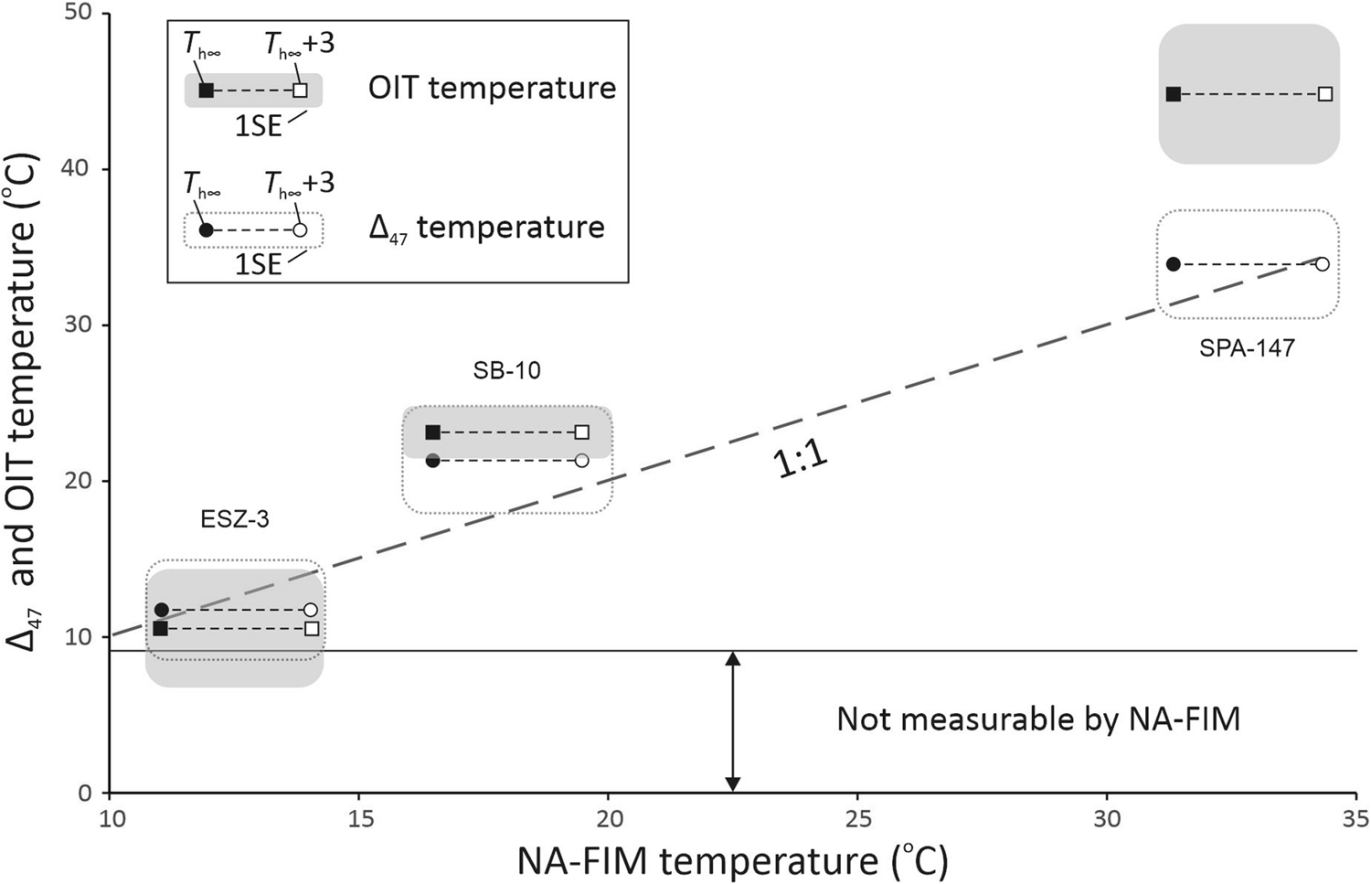
Comparison of NA-FIM, Δ_47_ and OIT temperatures (using the calibration by Daëron et al.^22^) obtained on the same samples. Black circles (rectangles) show measured T_*h∞*_; open circles (rectangles) show T_*h∞*_ + 3 °C (corrected for 300 m of hydrostatic pressure). Errors are shown at the by transparent (grey) boxes (1 SE, Table 1).

For these three samples robust estimates of δ^18^O_w_ were obtained by isotopic analysis of FI water (δ^2^H_w,_ δ^18^O_w_). Calcite formation temperatures were calculated using the fractionation equation of Daëron et al.^22^ (Table S4). A comparison of the NA-FIM, Δ_47_ and OIT temperatures is shown in Fig. 3. While temperature estimates obtained by the three methods in general agree for two samples, OIT yielded a significantly higher temperature than the other two methods for sample SPA-147 (Table 1, Fig. 3). Based on the relationship of Daëron et al.^22^ and measured Δ_47_ temperatures, we obtained a δ^18^O value of -14.4 ± 0.6 ‰ for the mineral-forming water, which corresponds to the measured and estimated δ^18^O_w_ using the FI isotope data (Table S4). Therefore, we propose that the δ^18^O_w_ may have remained rather constant over time, and the increase in δ^18^O_c_ across the calcite crystal (Fig. S2) may indicate a change in the temperature of the paleowater. The ~ 1.7 ‰ difference in δ^18^O between the bulk calcite crushed for OIT and the subsample measured for Δ_47_ thermometry could account for the higher T yielded by OIT.

### Assessing the precision and accuracy of temperature reconstructions

As discussed above, the NA-FIM method offers an internal quality check. As long as the measurements are done on FIAs rather than on individual inclusions, the consistency and reliability of the thermometric results is ascertained by the tightness of the T_*h*_ distribution. In FI research, a result is deemed consistent if 90% of the T_*h*_ measurements made on individual FIAs fall within a 10 °C-interval^7^. On the basis of this criterion, the data in this study are highly consistent, with some sets of measurements (T_*h∞*_) plotting within a 3–4 °C interval (Fig. 1). Such tight distributions essentially rule out any post-formational alteration of the initial volumes of FIs, either due to natural processes or sample preparation.

Temperatures derived by the NA-FIM and Δ_47_ methods define a linear relationship, justifying the application of linear regression analysis. For the evaluated Δ_47_ temperature calibration equation^19^, we obtained *R*^2^ = 0.91 (p-value < 0.001), if all data are considered. If NKQ4-3–1 and NKQ4-3–2 are treated as outliers, the calculated regression line has an *R*^2^ = 0.95 (p-value < 0.001). We therefore conclude that only 5% of the Δ_47_ temperatures are inconsistent with the NA-FIM temperatures. The standard error of the regression is ± 3.3 °C leading to a 95% confidence interval of ± 6.6 °C. This value is very similar to error margins of the individual clumped isotope measurements reported at the 95% CL (Table 2), supporting the notion of reporting Δ_47_ uncertainties at the 95% CL as suggested by previous studies (e.g. ^38^).

The observed deviations between NA-FIM and Δ_47_ temperatures do not show a systematic pattern. A similar non-systematic pattern was shown by an earlier study^4^ that focused on calcite cements formed at higher T (60–100 °C); however, FIM for these samples yielded much larger uncertainties (9–20 °C). For the spars examined in this study, these variations may be due to several processes that affect the Δ_47_ of calcite, including ^13^C–^18^O bond reordering during burial, and non-equilibrium isotope effects during mineral formation. Although ^13^C–^18^O bond reordering through solid-state diffusion^39^ may alter the initial Δ_47_ signal, isotope exchange in carbonate minerals requires T > ca. 100 °C and > 10^6^–10^8^ years^40^. As our samples formed below ~ 50 °C and experienced no later burial, the initial Δ_47_ values are regarded as pristine.

As noted above, one of the samples (NKQ4-3) may have been affected by non-equilibrium isotopic fractionation (e.g., rapid mineral precipitation and/or fast CO_2_ degassing) leading to temperature biases that would manifest in Δ_47_ temperatures being significantly higher than corresponding NA-FIM temperatures. We consider fast crystal growth highly unlikely as a potential cause of Δ_47_ disequilibrium given the large size of the spars (several cm) and their well-developed euhedral morphology (Sample selection in the Supplementary Information). In addition, no evidence of high supersaturation (e.g., instances of crystal nucleation; competitive growth of multiple crystals) was detected by optical microscopy. Dreybrodt^41^ suggested that in subaqueous settings, non-equilibrium isotope effects driven by Rayleigh distillation of the dissolved HCO_3_^−^ reservoir during degassing of CO_2_ and calcite precipitation can be excluded. Rayleigh distillation would lead to an increase in δ^18^O_c_ and a decrease in Δ_47,_ whereby a 1‰ shift in δ^18^O_c_ would correspond to ~ − 0.02‰ in Δ_47_^42,43^. Thus, we propose that processes other than CO_2_ degassing and fast crystal growth likely drove the measured disequilibrium Δ_47_ effects.

Although absolute temperatures differ between NA-FIM and clumped isotope thermometry in the two growth phases of sample NKQ4-3, a ca. 12 °C decrease is indicated by both NA-FIM data (from 32.7 ± 0.6 to 20.4 ± 0.5 °C) and Δ_47_ temperatures (from 42.5 ± 4.3 to 29.7 ± 3.0 °C, Table 1, Fig. 2 and Fig. S4a). Furthermore, δ^18^O_c_ increases from –11.9 to –9.4‰ from the core to the rim of this crystal (Fig. S2). This 2.5‰ rise also suggests a ca. 11 °C temperature increase^22^, assuming that the δ^18^O value of the paleowater remained unchanged, as suggested by the calculated δ^18^O_w_ values (Fig. S4b).

### Strengths and weaknesses of the three geothermometry methods for low-T calcite spar

The successful application of each of the three techniques (NA-FIM, Δ_47_-thermometry and OIT) depends on the sample material. These techniques are based on two features, the calcite matrix (Δ_47_-thermometry) and FIs therein (NA-FIM), and on the combination of these two (OIT).

NA-FIM appears to be the most robust and precise of the three methods for studying low-T calcite spar. It yields the smallest uncertainty (between 0.5 and 1.6 °C, Table 1) and thus allows for the reconstruction of fine-scale (< 3 °C) T changes (e.g., NKQ4-3 samples, Fig. S4). Furthermore, this method has a built-in quality check, defined by the tightness of T_h_ distributions.

Samples to be analyzed by NA-FIM must (i) contain primary FIs, (ii) the FIs must be sufficiently large to observe the phase transitions during microthermometric analyses, and (iii) the samples must have formed above 9 °C. Another caveat that needs to be considered is that FIM, when applied to calcite spar, provides minimum estimates of formation T only. For minerals formed at significant depth the pressure correction may reach several degrees. Since in most cases the depth of mineral formation is not well known, the pressure correction cannot be performed with high accuracy. Importantly, this constraint is not relevant for speleothems, which form under atmospheric pressure conditions.

Unlike NA-FIM, Δ_47_-thermometry can be performed on all calcite samples and it is applicable to T as low as 0°C^44^. Although this method can also detect T changes of less than 3–5 °C, individual measurements have larger analytical uncertainties when compared to NA-FIM. In our study of slow-growing calcite spar, 1 SE ranged from 1.5 to 3.8 °C (excluding the T uncertainty of the Δ_47_-T calibration after Anderson et al.^19^, Table 2), which may be further reduced by improved measurement statistics (e.g.^25,38^). Comparable uncertainties were obtained in a previous study^3^ for similar calcite spars formed at 10–20 °C and at 120–130 °C.

Finally, our study indicates that in addition to uncertainties discussed above, the ∆_47_ temperature deviate from NA-FIM temperature in a non-systematic way. The standard error of regression for the data is 3.3 °C, resulting in a 95% confidence interval of ± 6.6 °C.

The application of the OIT is rather challenging for calcite spar. Like the ∆_47_ method, OIT is based on the assumption that the calcite was formed in isotopic equilibrium with the paleowater. For samples formed in deep phreatic settings such as calcite spar, this assumption appears justifiable. It may be less so for calcite of vadose speleothems or travertine, whose formation is commonly associated with relatively fast degassing of CO_2_. In most cases, the question of whether a given sample was deposited in isotopic equilibrium remains unanswered, adding to the uncertainty of T estimates.

Furthermore, more than one equation describing the equilibrium isotope fractionation between water and calcite is available^22–24, 45–47^. The differences between OIT temperatures calculated in our study using various equations range between 7.0 and 10.2 °C (Supplementary Information, Table S4).

Independent knowledge of the δ^18^O value of the paleowater is crucial for the application of OIT. The most accurate approach is to determine this parameter by analysing the isotopic composition of FIs. However, this is only feasible for samples containing exclusively primary FIs. This is relatively common in speleothem calcite^25,48, 49^, but is rarely the case for calcite spar. For example, only six out of 13 of our samples fulfilled this criterion, and only three samples contained enough water for reliable analyses.

Further complication arises from the possibility of oxygen isotopic exchange between FI water and the host calcite. Such exchange is particularly likely if the mineral formed at T that are higher than the modern-day ambient T. In such situations, δ^18^O_w_ must be evaluated based on δ^2^H_w_ (which is not affected by isotopic exchange with the calcite host). This approach, however, introduces additional uncertainty because the relationship between δ^2^H_w_ and δ^18^O_w_ at a time of calcite formation must be assumed.

Finally, our limited data set shows a good agreement between OIT, NA-FIM and Δ_47_ temperatures for two samples (Fig. 3). In contrast, the third sample (SPA-147) shows a discrepancy between OIT and the other two geothermometry methods. This possibly arises from the much larger sample size required for OIT, resulting in an averaging of the formation T gradient captured by this sample (Fig. S1).

In summary, for future geothermometry work using low-T calcite spar, we recommend the use of NA-FIM if the calcite contains primary FIAs. Although this method is labour-intense, it provides the most precise and accurate T estimates of the three methods and it is insensitive to non-equilibrium isotopic fractionation. Furthermore, fine-scale (< 3 °C) T changes can be detected with NA-FIM, which is still challenging for Δ_47_-thermometry, given the larger analytical uncertainties of this method. Our study demonstrates that ∆_47_-thermometry provides reliable T estimates for calcite spars formed between 9 and 50 °C. Yet, these natural calcites might be affected by kinetic isotope fractionation, potentially leading to formation T uncertainties that may exceed 1 SE. Combining ∆_47_ with high-precision ∆_48_ measurements^44^ and continuing efforts in improving interlaboratory comparability are promising developments of future applications of low-T geothermometry using calcite spar.

## Materials and methods

### Sample preparation

Calcite spar samples were sectioned using a low-speed precision saw (IsoMet, Buehler). Doubly-polished 150–300 µm-thick sections were prepared at the University of Innsbruck for FI petrography and microthermometry. The positions of FIAs were marked both on the thick sections using a pen and on the original billets, to enable subsequent sampling for clumped isotope analysis. Based on petrographic observations, 13 FIA-bearing zones in 11 calcite samples were selected for this study. Two different areas were investigated in samples ESZ-2 and NKQ4-3 to check for potential T changes during the growth history of these crystals. Optical microscopy indicated no petrographic changes in ESZ-2, but distinct growth zones in NKQ4-3 marked by FI-rich areas suggest changing growth conditions. In NKQ4-3 the two studied FIAs are separated by a ca. 1 mm-thin red zone stained by iron oxides.

### Nucleation-assisted fluid-inclusion microthermometry (NA-FIM)

NA-FIM on single-phase FIs was carried out at the Department of Earth Science, University of Bergen. The analyses were performed using a Linkam THSMG 600 heating/freezing stage mounted on an Olympus BX53 microscope. The microscope is connected to an amplified Ti:sapphire femtosecond laser (CPA-2101, Clark-MXR, Inc.). The 775 nm laser beam is coupled into the microscope light path via a dual port intermediate tube equipped with a short-pass dichroic mirror and focused on the sample through a 100 × long-working-distance objective (Olympus LMPLFLN). Bubble nucleation was induced in the metastable liquid state of the inclusions by means of a single laser pulse^9^. The setup allows for repeated and precise *T*_*h*_ measurements of initially single-phase FIs. *T*_*h*_ was measured for each individual inclusion at least twice. The reproducibility (precision) is typically within 0.1 °C (± 0.05 °C). In addition, vapor bubble radii were measured at known T in order to correct for the effect of surface tension on liquid–vapor homogenization using the thermodynamic model proposed by Marti et al.^10^ The overall analytical precision of *T*_*h*_ for individual inclusions is in the range of ± 0.2 to 0.4 °C. Uncertainties of NA-FIM temperatures are given as standard error (1 SE) and also include analytical uncertainties.

Freezing experiments were performed at the University of Innsbruck. The measurements were carried out on a Linkam THMS600 heating-freezing stage mounted on an Olympus BX41 microscope. Fls were cooled down to −40 °C and then slowly heated to detect ice melting temperatures.

### Clumped isotope analyses of calcite

Clumped isotope analyses were carried out at the University of Heidelberg. In order to obtain a larger number of replicates, all samples except one (FEC-1) were re-analysed at the Isotope Climatology and Environmental Research Centre (ICER), Institute for Nuclear Research (ATOMKI), Debrecen (Tables 1 and 2). While few of the repeats were performed on the same powder samples on which the first set of measurements was obtained, most samples had to be re-drilled. Spar FEC-1 was not re-analysed as there was only very little material left.

#### Heidelberg

10–12 mg aliquots of calcite powder were obtained from the same parts of the samples where the FIs were studied. Each sample was split into several aliquots, and samples weighing 2–3 mg were used per measurement round. All aliquots were subject to identical preparation and measurement procedures. Clumped isotope analyses were carried out on a MAT 253 Plus isotope ratio mass spectrometer (Thermo Fisher Scientific) following the method described in Kluge et al.^50^ and Weise and Kluge^51^. All samples were reacted with phosphoric acid at 90 °C for 10 min in individual reaction vessels to produce CO_2_ for isotopic analysis. The CO_2_ was continuously collected during the reaction and cryogenically cleaned afterwards with an additional passage through a Porapak filled column at −35 °C. Each mass spectrometric analysis of the cleaned CO_2_ gas consisted of eight acquisitions with ten cycles per acquisition and included a background measurement for water vapour and air remainders^51^. Simultaneously to the *m/z* 44–49 readings a baseline signal on *m/z* 47.5 was recorded and used for pressure-baseline correction that affects *m/z* 47^51^.

For standardization, community-wide distributed carbonates (ETH1-4), Carrara marble, NBS 19, and equilibrated (5 °C, 90 °C) and heated gases (~ 1000 °C) were measured regularly.

#### Debrecen

12 samples (Tables 1 and 2) were measured at the Isotope Climatology and Environmental Research Centre (ICER), Institute for Nuclear Research (ATOMKI), Debrecen. The analyses were performed on a MAT 253 Plus isotope ratio mass spectrometer (Thermo Fisher Scientific), after phosphoric acid digestion at 70 °C using a Thermo Scientific Kiel IV automatic carbonate device. 100 μg aliquots of each carbonate sample measurement were replicated 12 times and measured alongside carbonate standards. ETH1, ETH2, and ETH3 were used as normalization standards, and IAEA-C2 was used as monitoring sample to determine the long-term reproducibility of the instrument (1 SD = 0.029‰; N = 57). Negative background, which is caused by secondary electrons on higher Faraday cup detectors, was corrected by applying a pressure-sensitive baseline (PBL) correction^52^ on all raw beam signals. Data evaluation, standardization, and analytical error propagation of Δ_47_ clumped-isotope measurements was carried out using the CO_2_ Clumped ETH PBL replicate analysis method, implemented in Easotope software^53^ using the revised IUPAC parameters for ^17^O correction^54^. Δ_47_ results are reported on the I-CDES-90 scale^55^.

The ∆_47_ values of the Heidelberg and Debrecen laboratories were combined by calculating an error-weighted mean of the (I)-CDES-90 values for each sample (Table 2). The ∆_47_ uncertainty of the combined data is given as standard error. Formation temperatures were calculated from the measured ∆_47_ values using the calibration curve of Anderson et al.^19^ that is related to an acid reaction temperature of 90 °C, avoiding additional uncertainties due to acid fractionation corrections. The temperature uncertainty of clumped isotope thermometry is reported as standard error (1 SE) and includes analytical and calibration uncertainty (95% CL) as provided by Anderson et al.^19^.

### Stable isotope analyses of calcite

Transects for stable isotope analyses were made at 1–3 mm increments. The powders obtained using a hand-held dental drill were analysed using a Delta V Plus isotope ratio mass spectrometer coupled to a Gasbench II (Thermo Fisher Scientific). The results are reported relative to the Vienna Pee Dee Belemnite standard (VPDB). The long-term precision of the measurements is 0.06‰ and 0.08‰ for δ^13^C and δ^18^O, respectively (1 SD)^56^.

### Stable isotope analyses of FI water

Calcite samples were crushed in a custom-built crushing device^57^ coupled to a Delta V Advantage isotope ratio mass spectrometer (Thermo Fisher Scientific) at the University of Innsbruck^17^. The isotopic composition of the released water was calibrated against in-house standards. For water amounts ranging between 0.1 and 1.0 µL the precision of the in-house water standard measurements was typically better than 1.5‰ for δ^2^H_w_ (1SD) and 0.8‰ for δ^18^O_w_. δ^18^O_w_ isotope values were also calculated from the measured δ^2^H_w_ using the Local Meteoric Water Lines for Austria^21^ (for SPA-147 and SB-10) and Debrecen, Hungary^20^ (for ESZ-3) and compared to measured δ^18^O_w_. Depending on the water content, 0.9 to 1.4 g of calcite was crushed for each measurement. The δ^2^H_w_ and δ^18^O_w_ isotope values are reported relative to the Vienna Standard Mean Ocean Water (VSMOW).

### Oxygen isotope thermometry (OIT)

Oxygen isotope values of FI water (δ^18^O_w_) and those of the host calcite (δ^18^O_c_) were used to calculate formation temperatures using the equation of Daëron et al.^22^. T estimates calculated by other equilibrium isotope fractionation equations are discussed in the Supplementary Information.

### Supplementary Information

Supplementary Information.

## Data Availability

All data generated or analysed during this study are included in this published article and its supplementary information files or is available from the first author upon request.
