# Supplementary material for: Geothermometry of calcite spar at 10–50 °C
Source: Sci Rep. 2024 Jan 18;14:1553. doi: 10.1038/s41598-024-51937-4 (PMC10796908; doi:10.1038/s41598-024-51937-4)
Supplement: Supplementary file 1 — Supplementary Information. [file 41598_2024_51937_MOESM1_ESM.pdf]

Supplementary information to

Geothermometry of calcite spar at 10–50°C

Gabriella Koltai[1*], Tobias Kluge[2,3], Yves Krüger[4], Christoph Spötl[1], László Rinyu[5], Philippe Audra[6], Charlotte Honiat[1], Szabolcs Leél-Őssy[7], Yuri Dublyansky[1]

[1]Institute of Geology, University of Innsbruck, Innrain 52, 6020 Innsbruck, Austria
[2]Institute of Environmental Physics, Heidelberg University, Im Neuenheimer Feld 229, 69120 Heidelberg, Germany
[3]Chair of Geochemistry and Economic Geology, Karlsruhe Institute of Technology, Adenauerring 20b, 76131 Karlsruhe, Germany
[4]Department of Earth Science, University of Bergen, Allégaten 41, 5007 Bergen, Norway
[5]Isotope Climatology and Environmental Research Centre (ICER), HUN_REN Institute for Nuclear Research (Atomki)
[6]Polytech´Lab, University of Nice, Côte d`Azur, 930 Route des Colles, Sophia-Antipolis, 06903 Nice, France
[7]Department of Physical and Applied Geology, Eötvös Loránd University, Egyetem tér 1-3, 1053 Budapest, Hungary

[*]Corresponding author: gabriella.koltai@uibk.ac.at

Sample selection: growth rates of calcite spars and isotopic equilibrium

Very slow-growing subaqueous calcite spar was collected in Hungary, France, Austria and Kyrgyzstan. The key features of these locations and the samples are summarized in Table S1.

In the context of this study, the slow growth rate of calcite spar is important as it suggests precipitation of calcite in isotopic equilibrium with the mineral-forming water and the absence of kinetic fractionation affecting $\delta^{18}O$ and clumped isotopes, which would affect paleotemperature calculations.

The calibration used in this study to calculate paleotemperatures by OIT is derived from natural calcite from Devils Hole (Nevada, USA) [1] and Laghetto Basso of Corchia Cave (Italy) [2], which grew extremely slowly (ca. 10^{-11} mmol cm^{-2} s^{-1};[2]). In contrast, laboratory growth experiments typically involve much faster rates of 10^{-9} to 10^{-7} mmol cm^{-2} s^{-1};[3–5]. Deviations of calibrations based on laboratory experiments from 'equilibrium' calibrations of Coplen[1] and Däeron et al.[2] can be explained by kinetic fractionation occurring in these experiments[4].

Samples studied in this project formed under stable conditions typical of deep phreatic environments, characterized by very low gradients of physical (P, T) and chemical parameters. The studied calcite spars are characterized by (a) large sizes up to 7 cm (individual crystals of up to 15-40 cm were reported from some of the sites studied[6]), (b) commonly perfectly developed euhedral crystal terminations, (c) a high degree of transparency, and (d) the absence of petrographic indications of growth irregularities, such as hiatuses or fluid inclusion-rich zones. Given these features, and considering general principles of crystal growth (e.g.,[7,8]), one may infer that growth of these spars occurred at very low degrees of supersaturation with respect to calcite.

Although the growth rates of the studied calcite spars cannot be determined (for all samples the ^{230}Th ages are in secular equilibrium, i.e. >600 ka BP), their growth rates were probably as low or lower than calcite from Devils Hole and Corchia Cave, given the presumed formation conditions.

From general physico-chemical considerations, the oxygen isotopic composition of calcite slowly precipitating from large volumes of water, where the dissolved inorganic carbon concentration remains constant in time, should be close to the value predicted by the Coplen[1] and Däeron et al. [2] equations[4]. This is because in water bodies with a depth exceeding 1 cm, isotope exchange with the surrounding atmosphere is negligible due to the very small coefficient of molecular diffusion of CO_2 ($2\cdot10^{-5}$ cm^2 s^{-1};[9]). This was empirically confirmed by studies of calcite precipitating in water pools in a Chinese travertine system[10]. Based on geological considerations, the depths of formation of spars studied in this project were greater than those in Corchia Cave (<1 m) and Devils Hole (60 m) on which the Coplen[1]–Däeron et al. [2] calibrations are based, as well as calcite from pools of a Chinese travertine system (10–40 cm;[10]), for which isotopic equilibrium precipitation was demonstrated.

In summary, we find that the conditions for the formation of the calcite spars examined were very stable and the growth rates were slow to ensure the deposition of calcite in isotopic equilibrium with the mineral-forming water.

Fluid-inclusion petrography

Primary FIs

Most primary single-phase FIAs are associated with inclusion-rich growth zones following crystallographically defined directions (e.g., parallel to crystal faces). Three-dimensional clusters of primary FIs are also common in some samples (Table S2).

Primary FIs show a variety of morphologies ranging from negative crystals to highly irregular shapes, while some are elongated along the crystallographic growth direction (Table S2). Four characteristic morphologies of primary FIs are present indicating the direction of crystal growth. Thorn-shaped (Fig. S1a), thorn-shaped with bulbous origin (Figs. S1b and c), and elongated inclusions (Figs. S1c and d) are usually oriented parallel to the c axis of the host crystal (Table S2). The fourth type shows stepped inclusion walls recording the advancement of individual growth layers during crystal growth (Figs. S1e and f).

Secondary and indeterminate FIs

Secondary FIs are mostly confined to healed fractures and cleavage planes and appear as planar arrays showing cross-cutting relationships with crystal growth zones (Fig. S1a). Secondary inclusions are often thin and flat and show necking-down. These inclusions indicate healing of thin cracks after the crystallization of the host mineral. Single-phase inclusions of indeterminate origin were also observed.

Isotope analyses

FI water isotopes and isotope geothermometry

Details on fluid inclusion stable isotope data and calculated $\delta^{18}O_w$ - $\delta^{18}O_c$ temperatures are given in Tables S4 and S5. For the samples for which robust estimates of $\delta^{18}O_w$ were obtained by isotopic analysis of fluid inclusion water, formation temperatures were calculated using among others the Däeron et al.[2], Kim and O'Neil[11], Demény et al.[12], Tremaine et al.[13] equations (Table S5).

Δ_{47} and calcite $\delta^{18}O$ and $\delta^{13}C$

The Δ_{47} measurements were performed on calcite powders in two different laboratories (see Methods). The difference between the obtained Δ_{47} values varies between 0.007 ‰ and 0.059 ‰, and no systematic offset was observed between the two laboratories (Table 2). The Δ_{47} values of all samples except for three spars (ESZ-3, NKQ4-3-1 and SB-10) agree within 1 SD. Part of this divergence may be attributed to measurement statistics and/or sample heterogeneity. For a normal distribution 68.3 % of the data fall within ± 1 SD. Thus, for a data set with 12 samples 3-4 samples are expected to deviate by more than the 1 SD, consistent with our data. It is also plausible that part of the difference is due to sample heterogeneity, as all three samples with deviations > 1 SD were re-drilled for clumped isotope analyses and therefore not exactly the same aliquots were analysed in the two laboratories.

As no systematic difference was found between the data obtained of the both laboratories, we averaged the results for each sample using uncertainty-based weighing. For samples with only two replicates (Heidelberg laboratory) we used the larger 1 SD for weighing. The calculated mean therefore takes into account the statistical significance of the measurements in both laboratories.

The stable oxygen and carbon isotopic composition of all calcite samples was measured along their growth axis to assess possible changes in growth conditions. These results are summarized in Fig. S2.

$\delta^{18}O_c$ values of the two different growth zones in sample ESZ-2 are identical. These two zones yielded identical Δ_{47} values within uncertainty (Table 1). In sample NKQ4-3, $\delta^{18}O_c$ values increase abruptly by 2.5 ‰ from the early to the late growth phase and Δ_{47} values also yield a 0.034 ± 0017 ‰ rise (Fig. S4).

A deviation towards less negative $\delta^{13}C$ values by about 5‰ is recorded by sample ESZ-3 (Fig. S2), which shows a high $\delta^{13}C$ variability along the growth axis (from -5.5 to 2.4 ‰). In contrast, the $\delta^{13}C$ values are lower and show a lower variability in samples ESZ-2 (-8.7 to -7.5 ‰) and RKC-2 (-8.9 to -5.3 ‰). $\delta^{18}O_c$ values of samples ESZ-2 and ESZ-3 are similar, while RKC-2 is characterized by lower values (Fig. S2). CO_2 degassing during calcite precipitation and concomitant isotopic evolution of the dissolved inorganic carbon could be the reason for this shift in carbon isotopes, which has also been observed in speleothems[14–16] and cave analogous laboratory experiments[17].

The combined assessment of Δ_{47} and calcite $\delta^{18}O_c$ and $\delta^{13}C$ values allows to detect isotopic deviations in samples from the same region. In our study, samples from the Pannonian Basin define a positive trend (Δ_{47}= 0.016x$\delta^{18}O$+0.84; R^2=0.86; Fig. S5a), as expected for a uniform water source. Δ_{47} values become larger with higher $\delta^{18}O_c$ values, as lower temperatures lead to larger Δ_{47} and to a larger $^{18}\alpha(CaCO_3\text{-}H_2O)$ fractionation[2] causing higher $\delta^{18}O_c$ values. Generally, Δ_{47} values become larger with more negative $\delta^{13}C$ values (Fig. S5b) and calcite $\delta^{13}C$ and $\delta^{18}O_c$ (Fig. S5c) values are significantly correlated (R^2=0.92, when ESZ-3 is excluded), suggesting that lower temperatures are likely related to a larger admixture of meteoric water with a correspondingly larger fraction of soil-derived CO_2 with very negative $\delta^{13}C$ values. Calcite precipitating from the hydrothermal endmember leads to a calcite $\delta^{13}C$ value of -1.2 ± 0.2 ‰, with calcite $\delta^{13}C$ becoming more negative (as low as -9.8 ± 0.2 ‰) close to present-day surface temperatures. Reconstructed water $\delta^{18}O$ values using the calibration of Kim and O'Neil[11] reveal a trend from -7.4 to -7.1 ‰ for the meteoric endmember and towards higher values for the hydrothermal endmember. The first endmember corresponds to the modern meteoric water with $\delta^{18}O$ values ranging from -25.1 ‰ to 0.0 ‰ in the Pannonian Basin (weighted annual mean of -9.6 ‰ VSMOW[18]).

References

1. Coplen, T. B. Calibration of the calcite–water oxygen-isotope geothermometer at Devils Hole, Nevada, a natural laboratory. *Geochim. Cosmochim. Acta* **71**, 3948–3957 (2007).

2. Daëron, M. *et al.* Most Earth-surface calcites precipitate out of isotopic equilibrium. *Nat. Commun.* **10**, 1–7 (2019).

3. Dietzel, M., Tang, J., Leis, A. & Köhler, S. J. Oxygen isotopic fractionation during inorganic calcite precipitation - Effects of temperature, precipitation rate and pH. *Chem. Geol.* **268**, 107–115 (2009).

4. Dreybrodt, W. Kinetic fractionation of the isotope composition of ^{18}O, ^{13}C, and of clumped isotope $^{18}O^{13}C$ in calcite deposited to speleothems. Implications to the reliability of the ^{18}O and Δ_{47} paleothermometers. *Acta Carsologica* **48**, (2019).

5. Hansen, M., Scholz, D., Froeschmann, M.-L., Schöne, B. R. & Spötl, C. Carbon isotope exchange between gaseous CO2 and thin solution films: Artificial cave experiments and a complete diffusion-reaction model. *Geochim. Cosmochim. Acta* **211**, 28–47 (2017).

6. Dublyansky, Y. *et al.* Hypogene Karst in the Tyuya-Muyun and the Kara-Tash Massifs (Kyrgyzstan). in *Hypogene Karst Regions and Caves of the World* (eds. Klimchouk, A., Palmer, A. N., De Waele, J.,

Auler, A. S. & Audra, P.) 495–507 (Springer, 2017).

7. Sunagawa, I. Characteristics of crystal growth in nature as seen from the morphology of mineral crystals. *Bull. Minéralogie* **104**, 81–87 (1981).

8. Sunagawa, I. *Crystals: Growth, Morphology and Perfection*. (Cambridge University Press, 2005).

9. Dreybrodt, W., Hansen, M. & Scholz, D. Processes affecting the stable isotope composition of calcite during precipitation on the surface of stalagmites: Laboratory experiments investigating the isotope exchange between DIC in the solution layer on top of a speleothem and the CO_2 of the cave atmosphere. *Geochim. Cosmochim. Acta* **174**, 247–262 (2016).

10. Yan, H., Sun, H. & Liu, Z. Equilibrium vs. kinetic fractionation of oxygen isotopes in two low-temperature travertine-depositing systems with differing hydrodynamic conditions at Baishuitai, Yunnan, SW China. *Geochim. Cosmochim. Acta* **95**, 63–78 (2012).

11. Kim, S.-T. & O'Neil, J. R. Equilibrium and nonequilibrium oxygen isotope effects in synthetic carbonates. *Geochim. Cosmochim. Acta* **61**, 3461–3475 (1997).

12. Demény, A., Kele, S. & Siklósy, Z. Empirical equations for the temperature dependence of calcite-water oxygen isotope fractionation from 10 to 70°C. *Rapid Commun. Mass Spectrom.* **24**, 3521–3526 (2010).

13. Tremaine, D. M., Froelich, P. N. & Wang, Y. Speleothem calcite farmed in situ: Modern calibration of $\delta^{18}O$ and $\delta^{13}C$ paleoclimate proxies in a continuously-monitored natural cave system. *Geochim. Cosmochim. Acta* **75**, 4929–4950 (2011).

14. Daëron, M. *et al.* $^{13}C^{18}O$ clumping in speleothems: Observations from natural caves and precipitation experiments. *Geochim. Cosmochim. Acta* **75**, 3303–3317 (2011).

15. Kluge, T. & Affek, H. P. Quantifying kinetic fractionation in Bunker Cave speleothems using Δ_{47}. *Quat. Sci. Rev.* **49**, 82–94 (2012).

16. Affek, H. P. *et al.* Accounting for kinetic isotope effects in Soreq Cave (Israel) speleothems. *Geochim. Cosmochim. Acta* **143**, 303–318 (2014).

17. Hansen, M., Kluge, T. & Scholz, D. Investigation of disequilibrium clumped isotope fractionation in (speleothem) $CaCO_3$ with cave analogous laboratory experiments using thin films of flowing solution. *Geochim. Cosmochim. Acta* **321**, 244–264 (2022).

18. Bottyán, E., Czuppon, G., Weidinger, T., Haszpra, L. & Kármán, K. Moisture source diagnostics and isotope characteristics for precipitation in east Hungary: implications for their relationship. *Hydrol. Sci. J.* **62**, 2049–2060 (2017).

19. Turgentin, E. Geological setting of the iron mine at the Esztramos Hill. *Topogr. Mineral. Hungáriáé* **V**, 37–50 (1997).

20. Takácsné Bolner, K. Fecske-lyuk. in *Magyarorszag fokozottan vedett barlangjai* (ed. Székely, K.) 986–188 (Mezőgazda Lap-és Könyvkiadó, 2003).

21. Dublyansky, Y., Spötl, C. & Steinbauer, C. Stegbachgraben, a mineralized hypogene cave in the Grossarl valley, Austria. *Hypogene Speleogenes. Karst Hydrogeol. Artesian Basins* **1**, 117–120 (2009).

22. Spötl, C. *et al.* Stable isotope imprint of hypogene speleogenesis: Lessons from Austrian caves.

Chem. Geol. **572**, 120209 (2021).

23. Audra, P. Hypogene Caves in France. in *Hypogene Karst Regions and Caves of the World* (eds. Klimchouk, A., N. Palmer, A., De Waele, J., S. Auler, A. & Audra, P.) 61–83 (Springer International Publishing, 2017).

24. Spötl, C., Mangini, A., Bums, S. J., Frank, N. & Pavuza, R. Speleothems from the High-Alpine Spannagel Cave, Zillertal Alps (Austria). in *Studies of Cave Sediments* (eds. Sasowsky, I. D. & Mylroie, J.) 243–256 (Springer US, 2004).

25. Hager, B. & Foelsche, U. Stable isotope composition of precipitation in Austria. *Austrian J. Earth Sci.* **108**, 2–13 (2015).

26. Vodila, G., Palcsu, L., Futó, I. & Szántó, Z. A 9-year record of stable isotope ratios of precipitation in Eastern Hungary: Implications on isotope hydrology and regional palaeoclimatology. *J. Hydrol.* **400**, 144–153 (2011).

27. Friedman, I. & O`Neil, J. R. *Compilation of stable isotope fractionation factors of geochemical interest*. (US Geological Survey Professional Paper, 1977).

Supplementary Figures

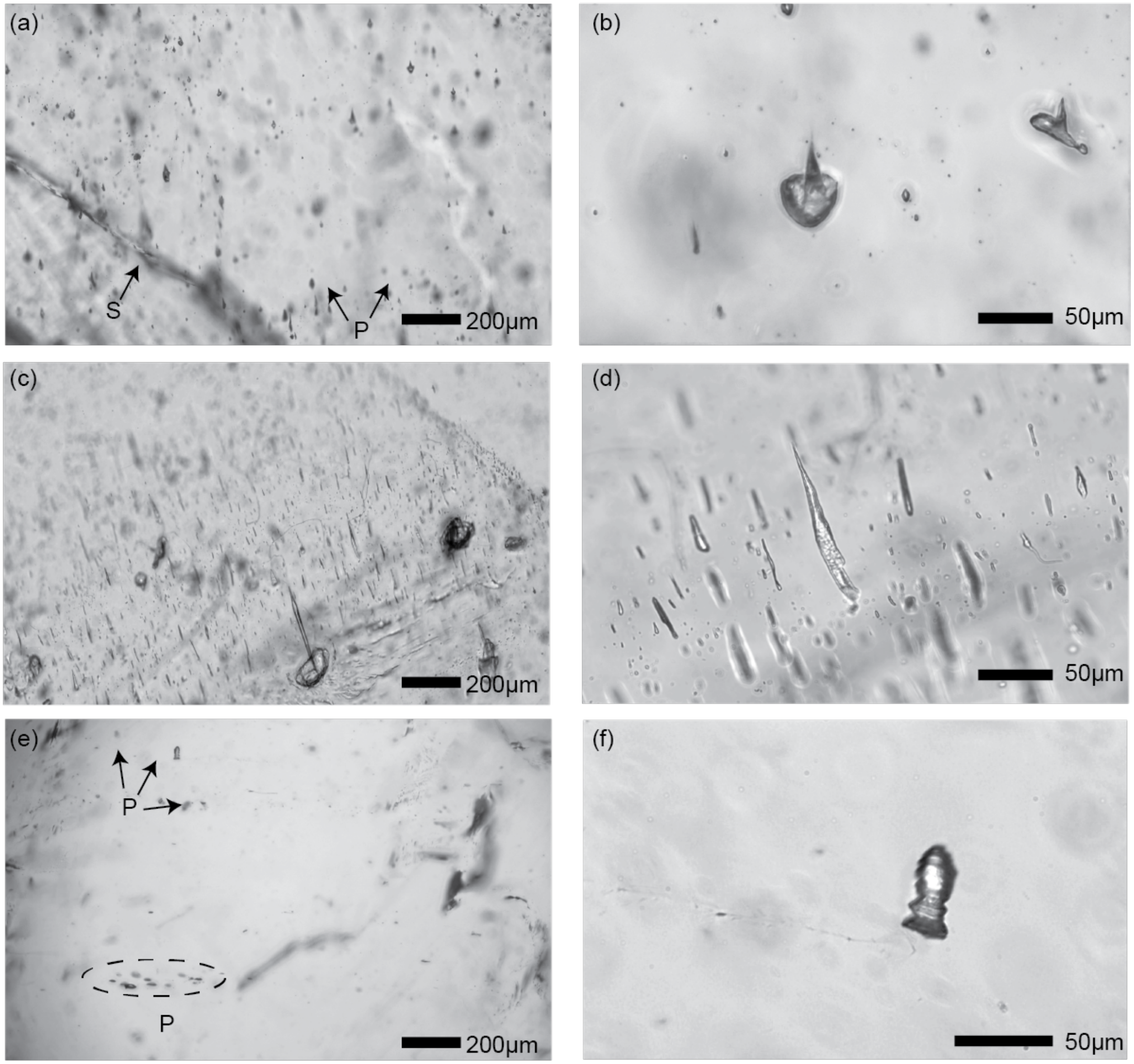

Figure S1. Typical appearance of primary FIs in the studied calcite spars. Primary FIAs (P) appear along former growth zones in (a and c), whereas secondary FIAs (S) occupy healed fractures. Primary FIs most commonly show thorn-shape (a), piriform (b and c) and elongated (c and d) morphologies. Primary FIs (P) appear isolated either in 3-dimensional patches or solitary (e) and can have stepped inclusion walls (f).

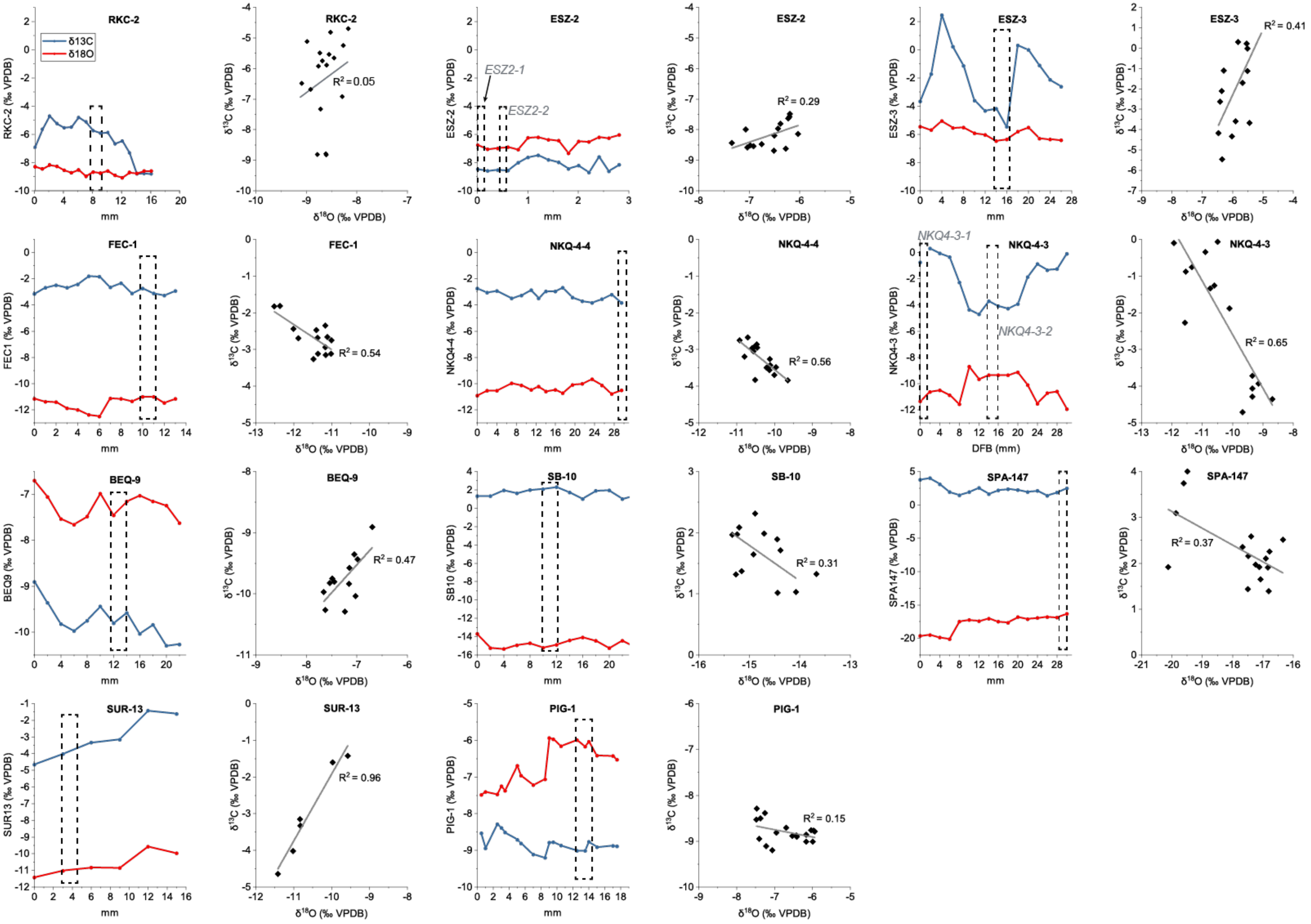

Figure S2. Stable isotope variability of the calcite spars ($\delta^{13}C$ values in blue, $\delta^{18}O$ values in red). Distance is shown from the base of each crystal. Dashed rectangles mark the (projected) positions of samples taken for FIM and Δ_{47}-thermometry. Note different scales of the y axes.

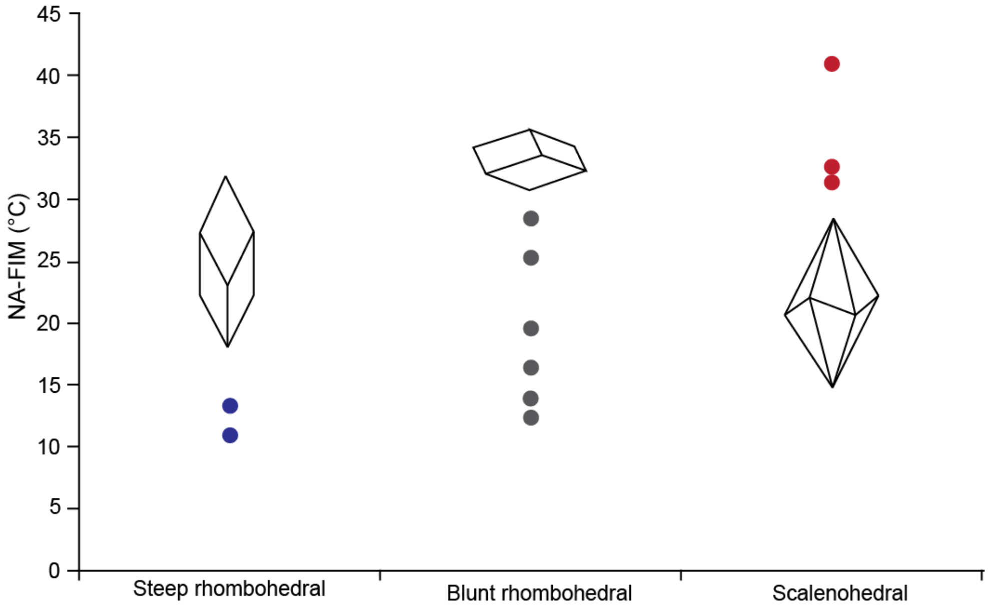

Figure S3. Comparison of formation temperatures (NA-FIM) and calcite crystal morphologies.

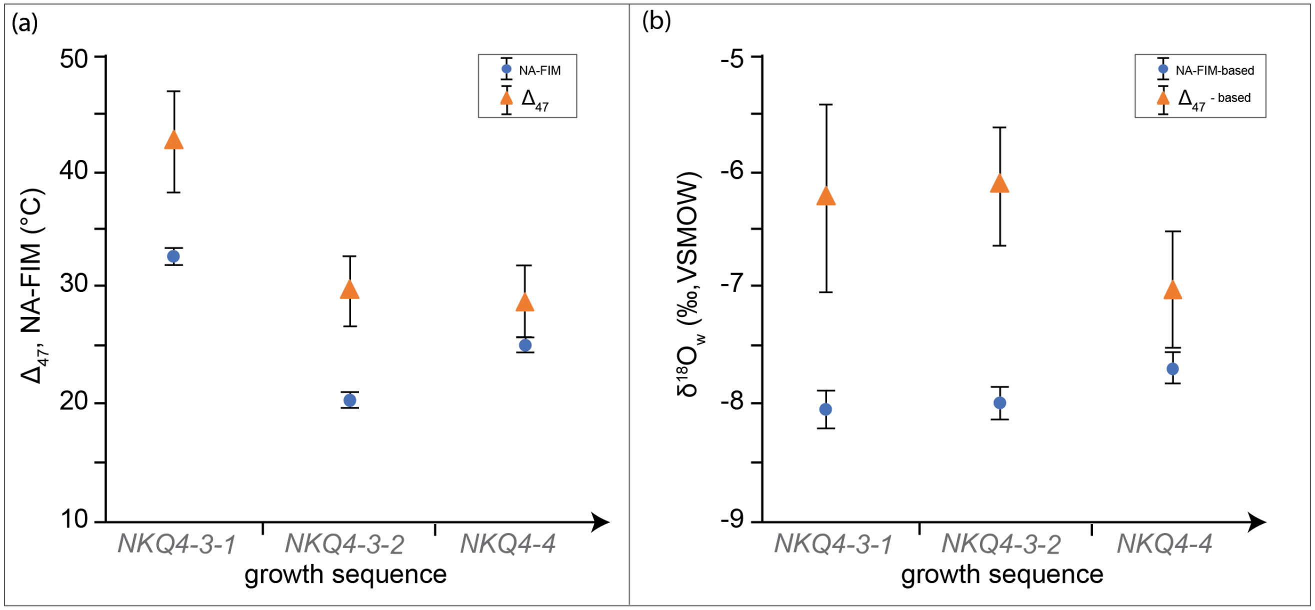

Figure S4. Comparison of formation temperature estimates by NA-FIM and Δ_{47}-thermometry for the NKQ samples. (a) Both thermometry methods indicate first a decrease (from sample NKQ4-3-1 to NKQ4-3-2), and NA-FIM then indicates an increase (from sample NKQ4-3-2 to NKQ4-4) in the temperature of the paleowater. This final increase is not resolved by the Δ_{47}-thermometry data probably due to their comparably larger uncertainties. (b) Calculated $\delta^{18}O$ of the paleofluid ($\delta^{18}O_w$) based on NA-FIM and Δ_{47} temperatures.

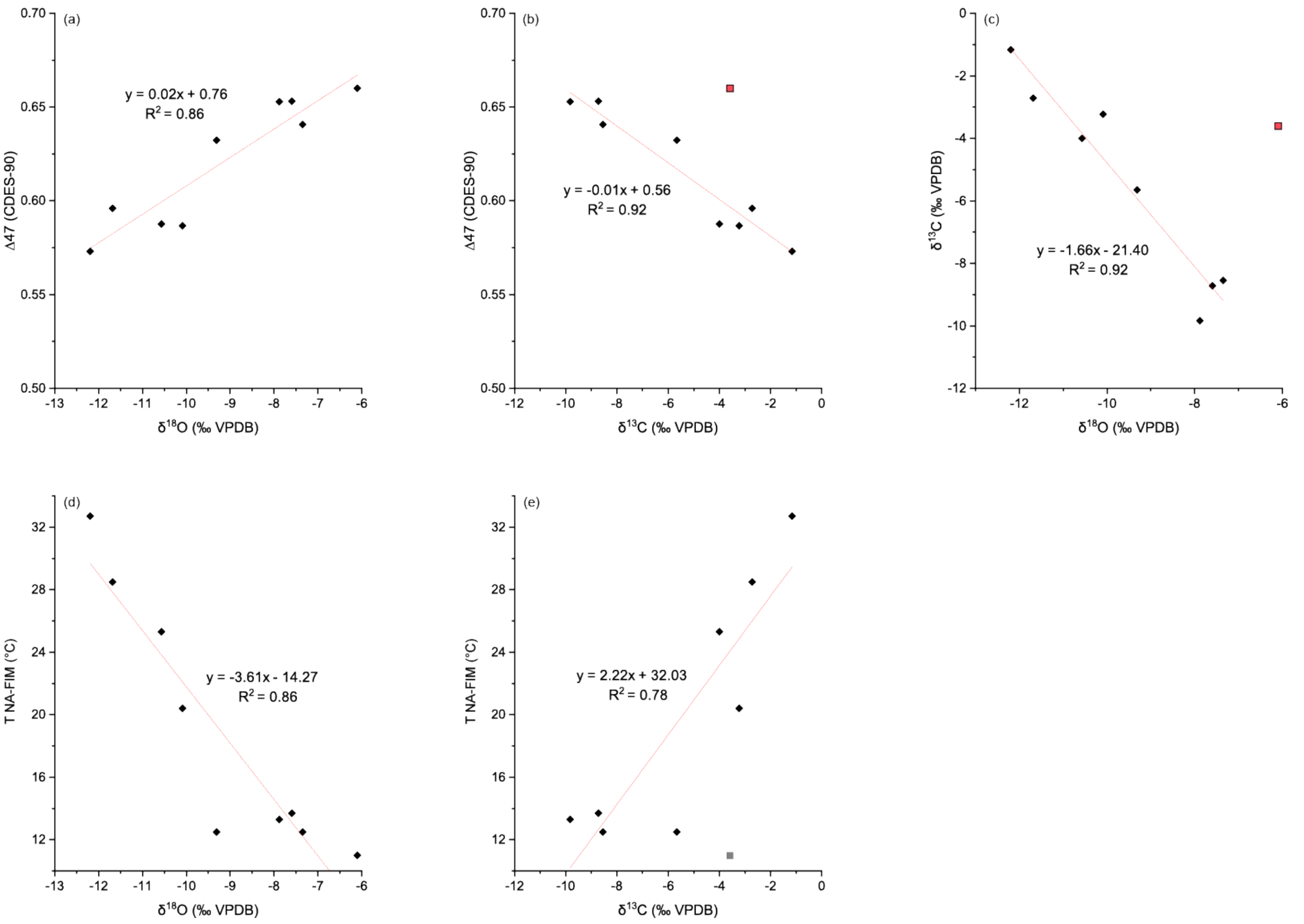

Figure S5. Correlation between calcite $\delta^{18}O$ and $\delta^{13}C$ values and Δ_{47} and minimum formation temperature estimates derived from NA-FIM of calcites spar collected in the Pannonian Basin (samples ESZ-2-1, ESZ2-2, ESZ-3, RKC-2, BEQ-9, NKQ4-3-1, NKQ4-3-2, NKQ4-4, FEC-1). The $\delta^{13}C$ values of sample ESZ-3 (red square) were excluded from the regression line calculations.

Supplementary Tables

Table S1. Calcite spar samples examined in this study.

Sample name	Site	Setting	Host rock	Calcite crystal habit	Mineral association	Age	Association with modern water**	Reference
ESZ-2	Esztramos quarry, Esztramos, Hungary	Grab sample of vein or vug calcite in mining talus	Steinalmi Fm (Middle Triassic limestone)	rhombohedral	carbonate hydroxyapatite, goethite, birnessite	> 600 ka* (based on RKC-2) Pliocene	Thermal springs in Bódva valley (16°C)<br>Karst water in Rákóczi caves (11°C)	Turgentin[19]
ESZ-3	Esztramos quarry, Esztramos, Hungary	Grab sample of vein or vug calcite in mining talus	Steinalmi Fm (Middle Triassic limestone)	steep rhombohedral	carbonate hydroxyapatite, goethite, birnessite	>600 ka* (based on RKC-2) Pliocene	Thermal springs in Bódva valley (16°C)<br>Karst water in Rákóczi caves (11°C)	
RKC-2	Rákóczi Cave, Esztramos, Hungary	Small cavity intersected by mine adit	Steinalmi Fm (Middle Triassic limestone)	rhombohedral	carbonate hydroxyapatite, goethite, birnessite	>600 ka* Pliocene	Thermal springs in Bódva valley (16°C)<br>Karst water in Rákóczi caves (11°C)	
BEQ-9	Berva quarry, Bükk Mts, Hungary	Cavity intersected by quarrying	Berva Fm. (Middle Triassic limestone)	steep rhombohedral	hydroxyapatite goethite marcasite hematite in other	>600 ka*		
FEC-1	Fecskelyuk, Bükk Mts., Hungary	Calcite crystals up to 10 cm in a hypogene cave	Bükkfensiki Fm. (Middle Triassic limestone)	rhombohedral		>600 ka* Miocene	Thermal spring nearby (~2.5km) in Miskolctapolca cave (28-30°C)	Takácsné[20]
NKQ4-3	Nagykőmázsa quarry, Bükk Mts., Hungary	Large fracture (1.5 m wide) lined by	Bükkfensiki Fm. (Middle	Split growth with multiple terminations,			Thermal spring nearby in	

		multiple generations of calcite	Triassic limestone)	resemble blunt rhombohedra			Miskolctapolca cave (28-30°C)	
NKQ4-4	Nagykőmázsa quarry, Bükk Mts., Hungary	Large fracture (1.5 m wide) lined by multiple generations of calcite	Bükkfensiki Fm. (Middle Triassic limestone)	Scalenohedral			Thermal spring nearby (~2.5km)in Miskolctapolca cave (28-30°C)	
SB-10	Stegbachgraben Grossarl Valley, Austria	Multiple generations of wall-lining calcite in a hypogene cavity	Klammkalk Fm. (Jurassic mylonitic calcite marble)	rhombohedral			Lukewarm springs in nearby Liechtenstein gorge (13.8-14.9°C)	Dublyansky et al.[21,22], Spötl et al.[24]
PIG-1	Pigette cave, Alpes-de-Haute-Provence, France	Calcite lining hypogene cave wall	Lower Jurassic marly limestone?	rhombohedral	traces of barite and secondary glauconite	Pliocene	Thermal sulfuric acid spring of Gréoux-les-Bains (42°C)	Audra[23]
SPA-147	Mitterschneidkar-Durchgangshöhle, Zillertal Alps, Austria	Solutional cavity lined with calcite, exposed by erosion	Hochstegen Fm. (Upper Jurassc calcite marble)	scalenohedral		>600 ka	Spring in nearby Hintertux valley at 1500 m a.s.l. (12-22°C)	Spötl et al.[22,24]
SUR-13	Surprise cave Tyuya-Muyun, Kyrgyzstan	Large geodes lined with calcite	Middle Paleozoic limestone	scalenohedral	barite	> 600 ka* Pre-Oligocene	Springs in nearby Dangi adit (ca. 20°C)	Dublyansky et al.[6]and unpublished data by the authors

Notes: *unpublished data by the authors; ** spatial association with modern water does not imply genetic relationship between calcite and this water.

Table S2. Petrographic characteristics of the main types of primary single-phase fluid inclusions (FI) and fluid inclusion assemblages (FIA).

Sample ID	FIA occurrence	FI shape	FI volume (µm³)
ESZ-2-1 core	GZ	TH, SW	1310-27050
ESZ2-2 rim	GZ	TH, SW	5700-155000
ESZ-3	GZ	NC, TH	4240-113890
RKC-2	GZ, P	TH, EG, SW	2290-94160
BEQ-9	GZ, P	EG, TH	4180-74610
FEC-1	IP, P	TH, SW	160-8460
NKQ4-3-1 core	P, GZ	R, IR	310-3280
NKQ4-3-2 rim	P, GZ	EG, NC, IR	410-12000
NKQ4-4	P	R, IR	460-323330
SB-10	GZ, P	SW	1350-44910
SUR-13	IP	NC, Q	50-3940
SPA-147	P	NC	160-1200
PIG-1	P	SW	1130-473970

Notes: Fluid inclusion assemblage occurrence: GZ – in growth zones, IP – isolated patches, P – pervasive, Inclusion shape: EG – elongated, IR – irregular, NC – negative crystal, R – rounded, SW – stepped walls, TH – thorn-shaped.

Table S3. Measured mean final melting temperature of ice.

Sample ID	$T_{ice\ melt}$ (°C)	Salinity (wt% NaCl equivalent)	Number of inclusions
ESZ-2-2	-0.4	0.7	6
FEC-1	-0.3	0.5	2
NKQ4-4	-0.4	0.7	4
SB-10	-0.5	0.9	7

Table S4. Stable isotope data of fluid inclusion water and calculated oxygen isotope temperatures (OIT).

Sample ID	δ^2H_w (‰ VSMOW)	SD (‰ VSMOW)	$\delta^{18}O_w$ measured (‰VSMOW)	SD (‰ VSMOW)	$\delta^{18}O_w$ calculated (‰VSMOW)	SD (‰ VSMOW)	$\delta^{18}O_c$ (‰VPDB)	SD (‰ VSMOW)	OIT	
									T (°C)	SD (°C)
ESZ-3	-60.7	5.3	-8.5	1.7	-8.8	0.7	-6.0	0.1	10.5	3.8
SB-10	-102.6	2.6	-14.2	1.3	-14.1	0.3	-14.4	0.1	23.1	1.7
SPA-147	-103.2	5.5	-14.3	2.4	-14.2	0.7	-18.3	0.1	44.4	4.6

Notes: $\delta^{18}O_w$ values were calculated from measured δ^2H_w using the Local Meteoric Water Line for Austria[25], for SB-10 and SPA-147. We used the Local Meteoric Water Line for Debrecen (Hungary) defined by Vodila et al.[26] for ESZ-3. Temperatures were calculated using the equation of Däeron et al.[2]. SD is the standard deviation.

Table S5. Measured NA-FIM temperatures and calculated OIT temperatures by using the calibration equations (denoted by subscripts): Friedman and O'Neil[27], Kim and O´Neil[11], Coplen[1], Demény et al.[12] for travertines, Demény et al. for speleothems[12], Däeron et al.[2]

Sample ID	$T_{h\infty}$ (°C)	$T_{Friedman\ and\ O'Neil}$ (°C)	$T_{Kim\ and\ O'Neil}$ (°C)	T_{Coplen} (°C)	$T_{Demény\_TRAV}$ (°C)	$T_{Demény\_SPL}$ (°C)	$T_{Tremaine}$ (°C)	$T_{Däeron}$ (°C)
ESZ-3	11.0	6.9	4.4	11.4	8.6	5.9	8.6	10.5
SB10-3	16.5	16.9	15.0	23.0	21.0	18.2	20.9	23.1
SPA-147	31.4	35.8	34.2	44.4	42.0	38.8	43.6	44.4
